# Prime editing: Mechanism insight and recent applications in plants

**DOI:** 10.1111/pbi.14188

**Published:** 2023-10-04

**Authors:** Tien V. Vu, Ngan Thi Nguyen, Jihae Kim, Jong Chan Hong, Jae‐Yean Kim

**Affiliations:** ^1^ Division of Applied Life Science (BK21 Four Program), Plant Molecular Biology and Biotechnology Research Center Gyeongsang National University Jinju Korea; ^2^ Division of Life Science Gyeongsang National University Jinju Korea; ^3^ Nulla Bio Inc. Jinju Korea

**Keywords:** prime editing, CRISPR‐Cas, precise gene editing, precision plant breeding, synthetic biology

## Abstract

Prime editing (PE) technology utilizes an extended prime editing guide RNA (pegRNA) to direct a fusion peptide consisting of nCas9 (H840) and reverse transcriptase (RT) to a specific location in the genome. This enables the installation of base changes at the targeted site using the extended portion of the pegRNA through RT activity. The resulting product of the RT reaction forms a 3′ flap, which can be incorporated into the genomic site through a series of biochemical steps involving DNA repair and synthesis pathways. PE has demonstrated its effectiveness in achieving almost all forms of precise gene editing, such as base conversions (all types), DNA sequence insertions and deletions, chromosomal translocation and inversion and long DNA sequence insertion at safe harbour sites within the genome. In plant science, PE could serve as a groundbreaking tool for precise gene editing, allowing the creation of desired alleles to improve crop varieties. Nevertheless, its application has encountered limitations due to efficiency constraints, particularly in dicotyledonous plants. In this review, we discuss the step‐by‐step mechanism of PE, shedding light on the critical aspects of each step while suggesting possible solutions to enhance its efficiency. Additionally, we present an overview of recent advancements and future perspectives in PE research specifically focused on plants, examining the key technical considerations of its applications.

## Introduction

Plant breeding includes generating genetic variants and selecting favourable plant traits via traditional and modern techniques (Osnato, 2023). To achieve novel and usable traits, the plant's genetic codes must be modified via spontaneous or introduced mutations by natural incidence or artificial means (Breseghello and Coelho, 2013; Gao, 2021; Lorenzo *et al*., 2023). The demand for modification of a specific genome region that controls a trait is consistent among the breeding techniques. For conventional breeding via selecting visible characteristics, crossing among parental lines, or random mutagenesis by radiation or chemicals, breeders attempt to screen large populations of offspring for the desired but not unexpected traits (Chen *et al*., 2019). However, at the genome scale, those techniques are very imprecise due to the difficulties in monitoring the genetic flows solely by observing the visible phenotypes (Breseghello and Coelho, 2013; Gao, 2021; Vu *et al*., 2022a).

**Figure 1:**
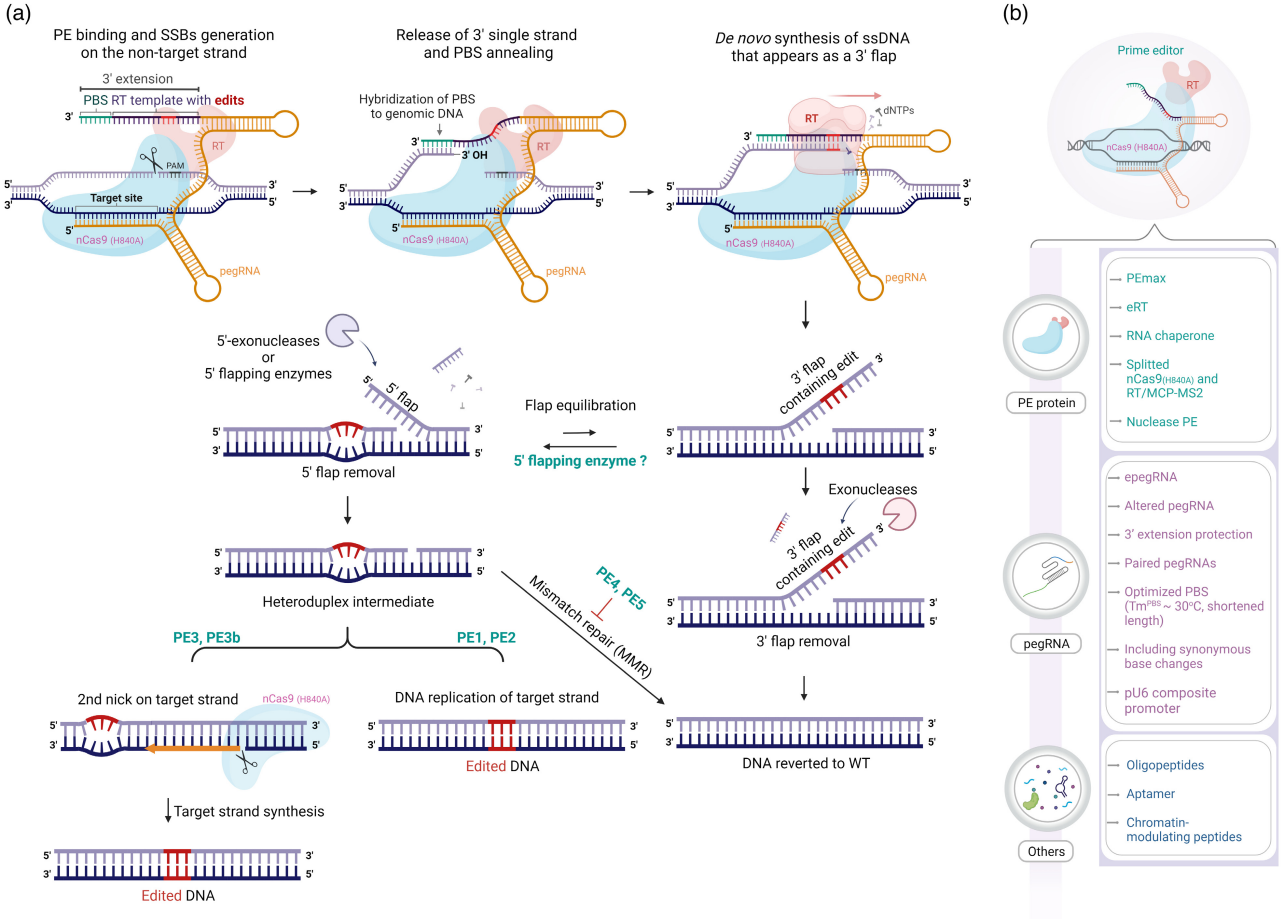
Overview of the PE mechanism. (a) Schematic diagram illustrating the components involved in the PE mechanism. The PE system comprises three main components: (1) nCas9 (H840A) proteins (faint blue colour), which induce single‐strand cleavage on the non‐target strand (light purple strand); (2) M‐MLV RT (pink colour), fused to nCas9 (H840A) via amino acid linkers, responsible for synthesizing a new single‐strand DNA incorporating desired edits (red coloured sequences); (3) pegRNA, containing a primer binding site (PBS) and an RT template that extends the 3′ end of a single‐guide RNA (sgRNA). The PE complex is guided to complementary sequences (target sequences) in the pegRNA spacer sequence. nCas9 (H840A) cleaves the nucleotide at the −3 position from the PAM site on the non‐target strand. The PBS (green‐coloured sequences) at the 3′ end of pegRNA binds to the complementary sequence on the non‐target strand, resembling a ‘primer’. M‐MLV RT initiates the synthesis of a new single‐strand DNA using the exposed 3′ hydroxyl group of the ‘primer’ and the RT template as a template. After synthesis, the newly synthesized single‐strand DNA competes with the original strand for insertion into the genome. If the original strand is preferentially installed in a 5′ flap while the new strand is removed, the existing genome sequence remains unchanged. Conversely, in a 3′ flap preference scenario, the new strand is preferentially installed while the original strand is removed, enabling desired edits to be introduced. Successful binding of the 3′ flap activates a mechanism to repair mismatched edit sequences. This can result in restoring the wild‐type (WT) sequence through the MMR pathway or the complete installation of the edit sequence by repairing the target strand. Additional cleavage of the target strand can trigger the installation of the edited strand. Green sequence: PBS; Red sequence: edit sequence; Faint blue protein: nCas9 (H840A); Pink protein: M‐MLV RT; Light purple strand: non‐target strand; Navy strand: target strand; Orange: spacer and scaffold of pegRNA. (b) Components of the PE system and various approaches that have enhanced prime editing efficiency.

Recently, the invention of an engineered CRISPR‐Cas9‐mediated genome editing (Jinek *et al*., 2012) has opened a new era for plant breeding. Importantly, this technology allows breeders to generate desired traits while keeping the genome's genetic code under precise control (Gao, 2021). Out of all the derivatives of the CRISPR‐Cas9‐based genome editing systems, the prime editor (PE) is unique, thanks to its characteristics in the editing process (Anzalone *et al*., 2019; Lin *et al*., 2020). PE employs a nickase SpCas9 (nCas9) that can generate an R‐loop at the target site and single‐stranded breaks (SSBs) on the non‐target strand (nCas9 (H840A) version) for binding to a prime editing guide RNA (pegRNA). The SSB of the non‐target strand releases a 3′ single‐stranded end that can anneal to the primer binding site (PBS) of a 3′ extension of the pegRNA that was pre‐designed to contain the PBS and reverse transcriptase (RT) template having the desired bases to be installed into the genome site (Figure 1). Then, an RT peptide fused to the nCas9 (H840A) acts on the primed heteroduplex by adding deoxynucleotides to the 3′‐OH of the nicked end corresponding to the code of the RT template. The *de novo* synthesized product appears as a 3′ flap that could be fixed into the genome via competition with the original sequence of the 5′ nicked end (Anzalone *et al*., 2019; Chen and Liu, 2023). The step may involve DNA damage repair pathways such as mismatch repair (MMR) (Figure 1) (Chen *et al*., 2021). Theoretically, PE can be used to instal various types of precise gene editing, such as all types of base substitutions, DNA sequence insertion and deletion. Moreover, the technique is efficient and precise in animals (Anzalone *et al*., 2019) and plants (Lin *et al*., 2020), though different loci often bring various efficiencies of editing.

Since the first report showing PE performance (Anzalone *et al*., 2019), the improvement and applications of PE tools in diverse organisms have brought much excitement into the field. All the original PE components have been recently modified and added with new features to achieve better efficiency (Chen and Liu, 2023). The PE complex was also used in pairs to facilitate more efficient short‐range editing as well as long‐range chromosomal fragment deletion, translocation and inversion (Anzalone *et al*., 2022; Jiang *et al*., 2022a; Kweon *et al*., 2023; Li *et al*., 2020b, 2023c; Lin *et al*., 2021; Molla *et al*., 2021; Tao *et al*., 2022a, 2022b). It was also used to insert recombinase recognition sequences to facilitate precise DNA sequence insertions at a large scale (Anzalone *et al*., 2022; Lin *et al*., 2021) and into carefully selected safe sites within the plant's genome (Sun *et al*., 2023a). Several recent data show improvements in PE in different aspects of its reaction (Chen and Liu, 2023). However, PE applications in plants have shown inconsistency among different loci and plant species and still require much effort to improve efficiency, especially in dicot plants (Chen and Liu, 2023; Jiang *et al*., 2022c; Lu *et al*., 2021; Vu *et al*., 2022b; Wang *et al*., 2021a). Therefore, this work aims to dissect the PE mechanism, update the recent advances in PE engineering and discuss several major technical issues to be considered to improve PE performance in plants. Finally, we provide our future perspectives on PE improvement and applications for precision plant breeding.

## Insight into the PE mechanism

### The original idea and its derivatives

The requirement of a highly efficient precision genome editing tool that can instal any desired changes to target DNAs was raised under the circumstances that base editing (BE) could be used to introduce only base transition and some types of base transversion (Anzalone *et al*., 2020; Chen and Liu, 2023) and CRISPR‐Cas‐based gene targeting (GT) needs to introduce homologous donor templates along with the molecular scissors (Anzalone *et al*., 2020; Chen and Liu, 2023; Fauser *et al*., 2012; Li *et al*., 2020b; Vu *et al*., 2020). Even today, not all types of base transversion are readily accessible with BEs (Chen and Liu, 2023). Moreover, the efficiency of GT was not sufficiently high at many loci (Guzmán‐Benito *et al*., 2023; Vu *et al*., 2019), and the introduced double‐stranded breaks (DSBs) may lead to unwanted impacts on the genome and the viability of targeted cells. Anzalone and coworkers thought that the 3′‐hydroxyl generated by nCas9 (H840A) could prime the RT reaction with a desired edit‐carrying extension of the single‐guide RNA (sgRNA) as the RNA template (Anzalone *et al*., 2019). The synthesized 3′ flap that carries the desired edit but does not fully match the genomic sequence could be fixed if the original, perfectly matched 5′ flap was removed by flapping enzymes or 5′‐exonucleases and subsequent DNA synthesis and repair occurred (Anzalone *et al*., 2019, 2020; Lin *et al*., 2020) (Figure 1). Considering the novelty and versatility of all types of precise base conversions, DNA sequence insertion and deletion (Anzalone *et al*., 2019, 2020; Chen and Liu, 2023; Lin *et al*., 2020) that can be installed in the genome, this was a great idea.

### Priming

**Figure 2:**
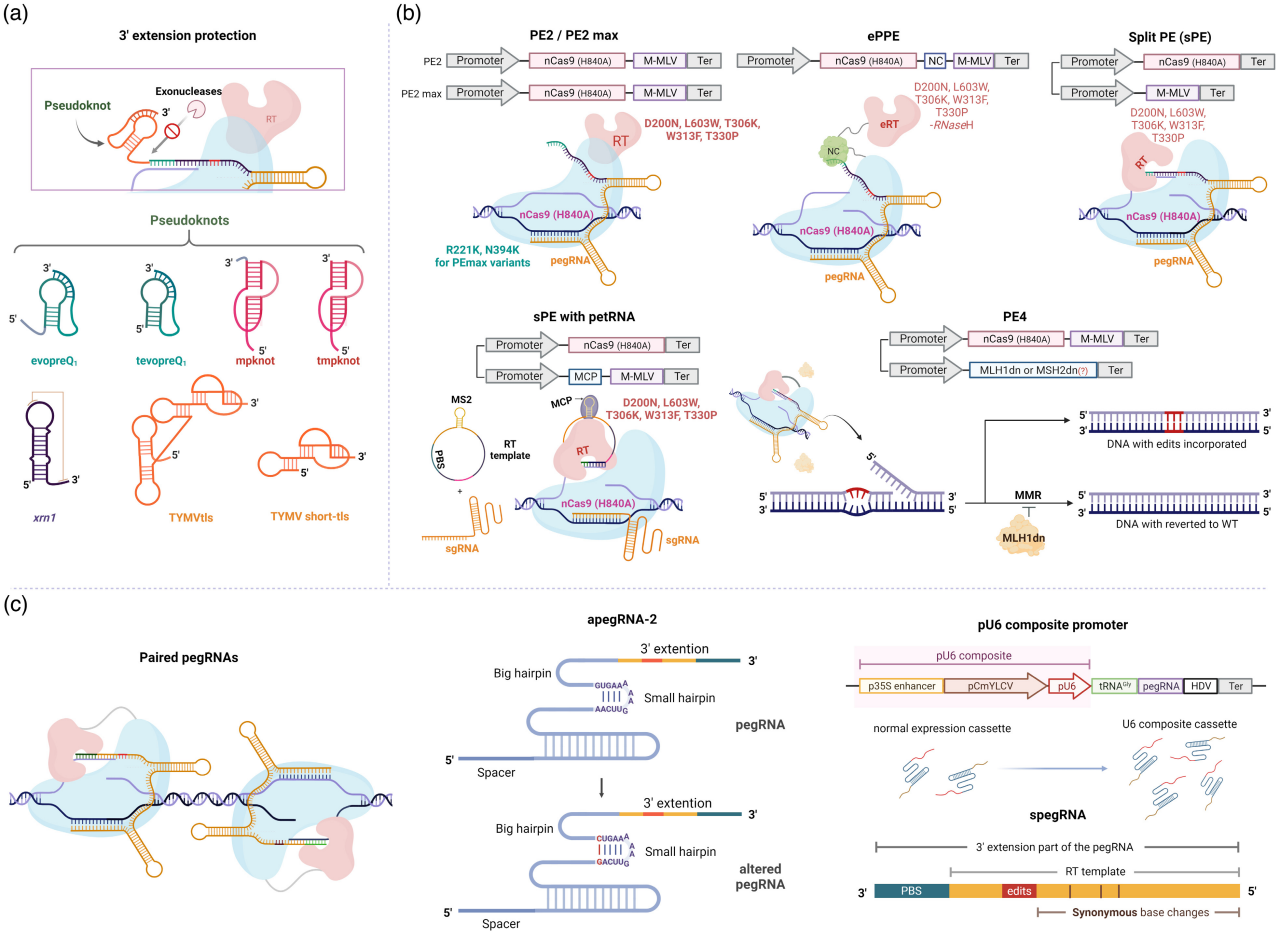
PE components and enhanced features. (a) Schematic representation of an approach involving 3′ extension protection by pseudoknots. Engineered pegRNA incorporates a pseudoknot structure at its 3′ end to safeguard the 3′ extension from exonuclease degradation. The secondary structures of several pseudoknot variants tested for enhancing prime editing efficiency are depicted. evopreQ1: modified prequeosine1‐1 riboswitch aptamer; tevopreQ1: evopreQ1 with a trimmed (grey at 5′ end) sequence; mpknot: frameshifting pseudoknot from M‐MLV; tmpknot: mpknot with a trimmed (grey at 3′ end) sequence; *xrn1*: Xrn1‐resistant RNA from sweet clover necrotic mosaic dianthovirus (SCNMV). (b) Protein‐based engineering strategies for PE. PE2: The original M‐MLV RT of PE1 is modified to enhance thermostability and DNA–RNA substrate affinity; PE2max: Contains additional modifications in nCas9 (H840A) to improve Cas9 nuclease activity. ePPE: Engineered Plant Prime Editor, where the NC is fused between nCas9 (H840A) for nucleic acid chaperone activity related to reverse transcription, and the ∆*RNase H* domain of M‐MLV is deleted to inhibit *RNase H*‐directed degradation of RNA–DNA heteroduplex. sPE: Separated expression of nCas9 (H840A) and M‐MLV RT for adeno‐associated viral (AAV) vector packaging capacity; petRNA: Split pegRNA into sgRNA and RT template‐PBS. The RT template‐PBS sequence is engineered into a circular form with the addition of the MS2 aptamer; sPE with petRNA: sgRNA guides the nCas9 (H840A)‐RT effector to the target site, and circularized petRNA is tethered to the MCP‐RT fusion protein by MS2. The PBS sequence of the tethered petRNA binds to a complementary sequence, and M‐MLV RT transcribes new single‐strand DNA using the RT template sequence. PE4: PE and MLH1dn are expressed independently. MLH1dn inhibits the MMR pathway, thereby increasing the probability of repair by the target strand. (c) pegRNA‐based strategies. Paired pegRNAs: The 3′ flap of two pegRNAs shares partial or complete complementary sequences. apegRNA‐2: the scaffold sequence of pegRNA is altered to stabilize the secondary structure by replacing a non‐C/G pair with a C/G pair in the small hairpin. RNA Pol III promoter: The Pol III promoter is combined with the widely used U6 promoter for sgRNA expression to enhance the transcription level of pegRNA. spegRNA: Introduction of synonymous mutations before or after the edit sequence to increase the efficiency of PE.

To prime a DNA polymerization reaction, a free 3′‐hydroxyl end should be available for forming a phosphodiester bond with an incoming nucleotide. During the replication of the genomic RNAs of retroviruses, the priming process is taken place by specific tRNA that exposes 3′‐OH substrate for polymerization by RTs (Harada *et al*., 1979; Marquet *et al*., 1995). Several published works show that the 3′ ssDNA broken end of the nontarget strand is released after cleavage by the CRISPR‐Cas9 complex and appears highly dynamic (Jiang *et al*., 2016; Richardson *et al*., 2016). The reverse transcription reaction is catalysed by a DNA polymerase that can use either the 5′ or 3′ extension of the Cas9 sgRNA as the template. The sgRNA‐3′ extension was a better pegRNA configuration than the 5′ one. The RNA extension is divided into two parts: the 3′ terminal part contains a sequence complementary to the 3′ nicked end of the cleaved DNA strand, which serves as the PBS. In this case, the 3′‐nicked terminal oligonucleotide acts as a primer for polymerization from its 3′‐hydroxyl end (Anzalone *et al*., 2019) (Figure 1a). Subsequently, the binding affinity of PBS to 3′‐nicked oligonucleotides was shown to affect PE performance, and the melting temperature (Tm) of the PBS was 30 °C for optimal PE in rice (Lin *et al*., 2021; Table 1). The intramolecular complementarity between the PBS and spacer sequences might also pose risks to the assembly of the PE protein and pegRNAs since the pegRNAs were shown to inhibit SpCas9's activities in tomato at various loci (Vu *et al*., 2022b). However, a similar observation was not revealed in human cells (Anzalone *et al*., 2019), possibly because of the differences in PE expression and delivery methods. Interestingly, the inherent autoinhibition of the PBS‐spacer complementarity mostly negatively affected PE used in the transfection of RNP with pegRNAs or plasmids using engineered pegRNA (epegRNA) that contain protected 3′ terminal pegRNAs and modified scaffolds (epegRNAs) (Nelson *et al*., 2022; Figure 2), and the impacts could be relieved by truncation of PBS (Ponnienselvan *et al*., 2023). However, it is still not clear why PE efficiency using the unprotected pegRNAs expressed from the plasmid was not inhibited, although the authors speculated that the unprotected 3′ terminal of the pegRNAs expressed from plasmids might be truncated, possibly by exonucleases, that mitigated the autoinhibition (Ponnienselvan *et al*., 2023).

**Table 1:** Major PE studies for precision gene editing in plants

No.	Species	Cas protein/RT enzyme*	Number of targeted loci/pegRNAs	Editing types	PBS length	RT template length	Editing efficiency range	Specific note	References
1	*Oryza sativa* and *Triticum aestivum*	nSpCas9 (H840A)/PE2	6 loci each, for rice and wheat/21 pegRNAs	All types of base substitution, short insertions and deletions	Varied, mostly 8–12 nt	Varied, locus‐dependent	PE2, PE3 and PE3b: up to 19.2% in rice and 1.4% in wheat (protoplast). PPE‐CaMV: up to 5.8% in rice (protoplast). At the plant stage, the editing frequency reached up to 21.8% in rice	PE3 and PE3b were not better than PE2. Insertion and deletion lengths reversely correlated with the editing efficiency. PE‐based deletion efficiency is higher than that of other edit types. PE performance at 37 °C was better than at 26 °C	Lin *et al*. (2020)
2	*Oryza sativa*	nSpCas9 (H840A)/PE2	6 loci/15 pegRNAs	Insertions and point mutations (except T to A)	Varied, mostly 10–16 nt	Varied, locus‐dependent, 10–25 nt range was tested	0.05%–1.55% in rice cells	PE2 and PE3 were comparable and required optimization. PE performance at 37 °C was not better than at 32 °C	Tang *et al*. (2020)
3	*Oryza sativa*	nSpCas9 (H840A)/PE2	1 loci/ 1 pegRNAs	Hexa base changes (4 point mutations)	13 nt	59 nt	2.22% at the plant stage	Only PE3 was tested	Li *et al*. (2020a)
4	*Oryza sativa*	nSpCas9 (H840A)/PE2	3 loci/6 pegRNAs	9 point mutations	8–14 nt	14–23 nt	0%–26% at the plant stage	Only PE3 was tested. Additional synonymous base changes introduced to the improved PE system highly enhanced PE efficiency	Xu *et al*. (2020a)
5	*Oryza sativa*	nSpCas9 (H840A)/PE2	3 loci/11 pegRNAs	Insertions and five point mutations	Varied, mostly 10–16 nt	Varied, locus‐dependent, 10–25 nt range was tested	0%–31.3% at the plant stage	Longer PBS (10–15 nt) and RT templates (10–34 nt) reduced and ultimately abolished editing at the OsACC1 locus. At the same locus, PE3 was not better than PE2	Xu *et al*. (2020b)
6	*Zea mays* and *Oryza sativa*	nSpCas9 (H840A)/PE2*	2 loci/3 pegRNAs	Several point mutations	10–13 nt	15–16 nt	0%–53.2% at the plant stage of maize. 0%–7.1% in rice protoplast	Enhancing the transcription of the pegRNAs by using 2× expression cassettes or CaMV35S + CmYLCV + U6 composite promoter with tRNA and HDV ribozyme enhanced PE activity in maize	Jiang *et al*. (2020)
7	*Oryza sativa*	nSpCas9 (H840A) and nSaCas9 (N580A)/PE2	5 loci/5 pegRNAs	G to A point mutation	13 nt	16 nt	Only working at the OsALS locus, one site (G to A), at 9.1% at the plant stage	PE3 was not better than PE2 with a GFP synthetic target. PE with nSaCas9 did not work at the ALS site	Hua *et al*. (2020)
8	*Oryza sativa*	nSpCas9 (H840A)/PE2	3 loci/3 pegRNAs	Insertion of AA and several point mutations	13 nt	20 nt	0.26%–2.04% at the callus stage. ~1.8% at the plant stage at the OsALS locus	PE3 did not increase editing efficiency. Using herbicides for selecting the PE events	Butt *et al*. (2020)
9	*Solanum lycopersicum*	nSpCas9 (H840A)/PE2	3 loci/7 pegRNAs	Insertions and some point mutations	14 nt	13–21 nt	0.025–1.66% from a mixture of 280 regenerated shoots. Up to 6.7% at the plant stage	Only PE3 was tested. No obvious phenotype was observed	Lu *et al*. (2021)
10	*Oryza sativa*	nSpCas9 (H840A) and nSpG‐Cas9 (H840A)/PE2	9 loci/15 pegRNAs	All types of base substitution, short insertions and deletions	5–17 nt. The Tm of PBS should be around 30oC	9–26 nt	Up to 24.5% with SpCas9 and up to ~3% with SpG in rice cells	The Tm of PBS should be around 30 °C, and using paired pegRNAs substantially enhanced PE efficiency	Lin *et al*. (2021)
11	*Nicotiana benthamiana*, *Oryza sativa* and *Arabidopsis thaliana*	nSpCas9 (H840A) and nSpG‐Cas9 (H840A)/PE2	1 locus in tobacco and rice; 2 loci in *Arabidopsis*. Total 4 pegRNAs	Insertion and several point mutations	13 nt	13–79 nt	0.06 ± 0.03% at a bacterial locus in tobacco leaves. 0.01%–2.2% at a genomic site in rice protoplasts. 0.07 ± 0.12% at a genomic site in *Arabidopsis* protoplasts	The 66‐bp insertion is the largest reported for PE and provides important proof of concept for fluorescent tagging using PE	Wang *et al*. (2021a)
12	*Oryza sativa*	nSpCas9 (H840A)/PE2	1 locus (*OsACC1*)/64 pegRNAs	Random triplex base substitutions	13 nt	10 nt	PE efficiency ranged from 0.01% to 8.53%	PE was used as a random mutagenesis tool for achieving herbicide‐tolerant alleles *in planta*	Xu *et al*. (2021b)
13	*Oryza sativa*	nSpCas9 (H840A); nSpCas9 (R221K/N394K/H840A) /PE2	17 loci/26 pegRNAs	Insertions, deletions and some point mutations	Varied	Varied	enpPE2 showed 64.58% to 77.08%. enpPE2 showed 25.60% (ACC‐T site), 11.50% (PDS‐T site), 33.75% (ALS‐T site) and 31.77% (CDC48‐T site)	Editing efficiency increased 2.35‐ to 29.22‐fold using epegRNAs with evopreQ1 modification (pegRNA‐evopreQ1)	Li *et al*. (2022b)
14	*Oryza sativa*	nSpCas9 (H840A)/PE2	6 loci/8 pegRNAs	Several point mutations	Varied	Varied	The double surrogate PE3 system could stimulate the PE efficiency up to ∼50‐fold compared with the conventional PE3	The surrogate system enables the PE of non‐editable targets such as *OsDHDPS* and *OsNR2*, which is otherwise impossible when using the original PE3	Li *et al*. (2022a)
15	*Oryza sativa*	nSpCas9 (H840A)/PE2/3	2 loci/10 pegRNAs	Various base substitutions, deletions and insertions	13 nt	10–16 nt	Up to 66.7% among the targets of two tested loci	The secondary structure of pegRNA might contribute to the variation in PE efficiency among loci	Li *et al*. (2022e)
16	*Oryza sativa*, *Triticum aestivum* and *Homo sapiens*	nSpCas9 (H840A)/PE2/ePPE	41 loci/83 pegRNAs	Insertions, deletions and some point mutations	Varied	Varied	The ePPE (‐RNase H domain) and NC combination synergistically enhanced the efficiency by, on average, 5.8‐fold compared with the original PPE in cell culture	Removing the RNase H domain further stabilizes the heteroduplex between the sgRNA–DNA and the nCas9–RT–pegRNA complex. The NC viral protein serves as a chaperone during the reverse transcription process via its nucleic acid‐annealing activities and interactions with the RT enzyme	Zong *et al*. (2022)
17	*Oryza sativa*, *Zea mays* and *Homo sapiens*	nSpCas9 (H840A)/PE2/ePPE	11 loci/15 pegRNAs (rice); 5 loci/7 pegRNAs (maize); 3 loci/3 pegRNAs (human cells)	All types of base substitutions	Varied	Varied	Up to 24.3% in rice, 6.2% in maize and 12.5% in human cells	N‐terminal fusion of RT to the nCas9 (H840A) was better than C‐terminal fusion. Multiple base substitutions within the RT template enhanced PE efficiency	Xu *et al*. (2022)
18	*Physcomitrium patens* and *Solanum tuberosum*	nSpCas9 (H840A)/PE2	3 loci/16 pegRNAs	Insertions, deletions and some point mutations	10–14 nt	15–19 nt	0.06% of the transformed protoplasts	PE3 does not improve significantly PE2 PE in *P. patens*	Perroud *et al*. (2022)
19	*Oryza sativa, Arachis hypogaea* L.*, Vigna unguiculata, Cicer arietinum* L.	nSpCas9 (H840A)/PE2	Synthetic target	A G‐to‐C base conversion activating GFP expression	N/A	N/A	0.2%–0.5% in peanuts, chickpeas and cowpeas	In most of the cells, only paired pegRNAs resulted in GFP activation	Biswas *et al*. (2022)
20	*Arabidopsis thaliana*	nSpCas9 (H840A)/PE2	3 loci/3 pegRNAs	Base substitutions (A‐ > T; G‐ > A; A‐ > G)	13–15 nt	10–17 nt	0.63%–1.44% in *Arabidopsis* cells	Optimization of the expression of PE proteins enhanced PE efficiency in *Arabidopsis*	Jiang *et al*. (2022b)
21	*Zea mays*	nSpCas9 (H840A)/ePE5max	4 loci/4 pegRNAs	Several base substitutions	13‐nt	16–27 nt	Up to 21.5%	ePE5max was better than ePE3max	Qiao *et al*. (2023)
22	*Physcomitrium patens*	nSpCas9 (H840A)/PE2	3 loci/16 pegRNAs	Insertions, deletions and some point mutations	10–14 nt	15–19 nt	0%–5%. The best efficiency was obtained with epegRNAs (knot)	The PE efficiency was significantly enhanced up to 143 folds by using the pUbi promoter to drive the expression of the PPE gene and epegRNAs	Perroud *et al*. (2023)
23	*Triticum aestivum*	nSpCas9 (H840A); nSpCas9 (R221K/N394K/H840A/)/PE2/ePPEplus	12 loci/18 pegRNAs	Base substitutions (C‐>T; G‐>T; C‐>G; C‐>A; A‐>C; G‐>C), several short insertions and deletions	N/A	N/A	Up to 74.5%	ePPEplus was created by adding V223A mutation to the eRT. Multiplexed PE was possible that simultaneously edited four to ten targets	Ni *et al*. (2023)
24	*Oryza sativa*	nSpCas9 (R221K/N394K/H840A/)/enpPE2/ePE2	10 loci/26 pegRNAs	Several types of base substitutions, deletions, insertions and protein tagging	Varied	Varied	Up to 70.54% efficiency with ePE2 for knocking‐in 6XHIS and HA tags. 8.33%–25% frequency of GRAND‐based 3XFLAG tag knock‐in	The tagging efficiency was locus‐dependent. GRAND‐based knock‐in (Table 2) significantly improved tagging efficiency	Li *et al*. (2023b)
25	*Oryza sativa*	nSpCas9 (H840A)/PE2 fused with T5 exonuclease	5 loci/6 single pegRNAs/8 dual‐pegRNAs	Several types of base substitutions, deletions and insertions	10–12 nt	12–25 nt	PE efficiency was enhanced from 1.7‐ to 2.9‐fold with T5 exonuclease N‐terminal fusion of nCas9 (H840A)	Dual‐pegRNAs improved PE efficiency	Liang *et al*. (2023)

*RT from PE2: M‐MLV RT (D200N/L603W/T330P/T306K/W313F).

Since the 3′ PBS‐RT template extension is the terminal sequence of the pegRNA, it may be vulnerable to nucleolytic damage by 3′–5′ exonucleases (Garneau *et al*., 2007; Ibrahim *et al*., 2008). However, the 3′ extension contains the terminal PBS critical for primer annealing and essential for initiating RT reactions. Shortening the PBS by spontaneous or enzymatic damages will lead to a reduction in PE efficiency. Therefore, adding protective sequences to the 3′ terminal of pegRNAs (Figure 2a) or circularizing the RNA template (petRNA) (Figure 2b) led to significant improvements in CRISPR‐Cas9's (Riesenberg *et al*., 2022; Rozners, 2022) and PE's (Li *et al*., 2022c; Liu *et al*., 2021b, 2022; Nelson *et al*., 2022; Zhang *et al*., 2022) activities as well. Interestingly, protected pegRNAs may also require a shorter PBS to mitigate the PBS‐spacer autoinhibition for better efficiency (Ponnienselvan *et al*., 2023). Further, protecting the nicked 3′ ssDNA end released from the nontarget strand by the dsDNA binding domain of Rad51 (Rad51 DBD) might help to enhance priming (Song *et al*., 2021). However, whether the Rad51 DBD enhanced PE by the proposed mechanism or by improving pegRNA binding to the nicked 3′ terminal sequence was unclear. In fact, the PE improvement of Rad51 DBD was higher when the melting temperature of the PBS sequences was lower, indicating a possible role of Rad51 DBD in increasing the binding affinity of PBSs (Song *et al*., 2021). In contrast, PE using Rad51 DBD in plants exhibited an even reduction in PE efficiency (Li *et al*., 2022b), indicating the approach requires further studies for plant applications.

Another strategy for enhancing priming was shown with additional truncated (Dahlman *et al*., 2015) proximal gRNAs (Chen *et al*., 2017) to the main pegRNAs that were believed to be able to relax the chromatin structure by binding but not activating the cleavage by the Cas protein (Park *et al*., 2021). Several chromatin‐modulating peptides (CMPs), such as high‐mobility group nucleosome binding domain 1 (HN1) and histone H1 central globular domain (H1G) (Ding *et al*., 2019), were also used by Park and coworkers for improving the PE system. Ultimately, the CMP‐PE‐V1 (with HN1‐nCas9‐H1G‐RT fusion configuration) returned the highest PE efficiencies at all the tested sites (Park *et al*., 2021). This strategy may be more helpful in enhancing the accessibility of the Cas complex to the targeted sites, especially those located within the heterochromatin regions (Ding *et al*., 2019; Park *et al*., 2021).

### RNA‐dependent DNA polymerization by the reverse transcriptase

An RNA‐dependent DNA polymerase or reverse transcriptase (RT) catalyses the production of complement DNA (cDNA) from single‐stranded RNA templates in a reaction called reverse transcription. They are used by retroviruses or retrotransposons to produce their genetic materials during invasion or transposition, respectively. RT enzymes were first described in a retrovirus that caused cancer in mice (Baltimore, 1970; Temin and Mizutani, 1970) and usually possess activities of RNA‐dependent DNA polymerase, RNase H and DNA‐dependent DNA polymerase (Roth *et al*., 1985; Tanese and Goff, 1988). The RT enzyme can polymerize from the 3′‐OH of the unpaired terminal nucleotide of a tRNAPro primer (Gilboa *et al*., 1979; Harada *et al*., 1979). The processivity of M‐MLV RT is robust enough to replicate the ~9‐kb genome of Moloney murine leukaemia virus (M‐MLV). The RT enzyme used in the PE2 configuration (Figures 1 and 2b) was from M‐MLV (Scolnick *et al*., 1970; Shinnick *et al*., 1981) that was modified with several point mutations (D200N/L603W/T330P/T306K/W313F) for enhancing thermostability and DNA–RNA substrate affinity (Anzalone *et al*., 2019). RT enzymes usually pause or stop polymerization at sites containing homopolymer nucleotides or stem‐loops. During PE processing, the RT reaction was primed by the terminal 3′‐OH of the nicked non‐target strand (Figure 1a) and might be stopped at or just before the stem‐loops of the sgRNAs. As a result, besides the precisely installed edits, there were byproducts containing nucleotides of the sgRNA scaffold, averaged at 1.7 ± 1.5% frequency of scaffold insertion (Anzalone *et al*., 2019). The inaccessibility of RT deep into the scaffold might also be due to the scaffold space constraint by nCas9 occupation or the efficient removal of mismatched 3′ flap ends that contained the scaffold sequences (Anzalone *et al*., 2019).

The synthesis of cDNA using RNA templates by RT enzymes is widely used by retroviruses during the replication of their genomes. The single‐stranded nature of the RNA templates may pose challenges to the progression of the enzyme since secondary and tertiary structures may arise from the RNAs (Harrison *et al*., 1998), especially when the RNA templates are lengthy. Retroviruses such as HIV use RNA chaperones to support the RT reaction. RNA chaperones work to unstabilize the secondary or tertiary structure, which helps to release RT enzymes from the stalled or paused state. In the PE process, adding an RNA chaperone from HIV called nucleocapsid (NC) protein (Figures 1b and 2b) improved PE performance in plants (Zong *et al*., 2022). In the same work, the authors showed that eliminating the RNase H domain from the RT enzyme (eRT) (Figure 2b) also helped enhance the prime editor's processivity. RNase H activity is required to degrade the tRNA primer (Omer and Faras, 1982) and the viral RNA template in RNA–DNA hybrids after the first minus cDNA strands have been completely synthesized (DeStefano *et al*., 1991; Mölling *et al*., 1971). Zong and coworkers hypothesized that deleting the RNase H domain may inhibit pegRNA degradation, enhancing PE activity (Zong *et al*., 2022; Figure 2a and Table 1). Alternatively, the RNase H‐deficient RT might be more stable and tightly bound to the primer–substrate complex (Gerard *et al*., 2002; Oscorbin and Filipenko, 2021) and thus improve the polymerization. Nevertheless, recent data indicates that the RNase H‐truncated RT variant might be the most suitable option for unstructured RT templates (Doman *et al*., 2023). In addition, Ni and coworkers recently showed that adding the V223A mutation to the eRT version further enhanced the plant (rice) codon‐optimized PE (PPE) (Lin *et al*., 2020) and engineered PPE (ePPE) (Jin *et al*., 2023; Zong *et al*., 2022) efficiency up to 33‐fold and 6.4‐fold, respectively, in wheat (Ni *et al*., 2023).

Besides M‐MLV RT, Lin and coworkers tested several RT enzymes, such as cauliflower mosaic virus (CaMV) or *E. coli* retron‐derived RT in PE experiments, in rice and wheat. However, none worked better than the M‐MLV RT (Lin *et al*., 2020). In fact, for enhancing the processivity of the M‐MLV RT enzyme in the first PE work, several point mutations (D200N, L603W, T306K, W313F and T330P) were introduced to it (Anzalone *et al*., 2019; Oscorbin and Filipenko, 2021) (Figure 2b). Therefore, there is still room for improvement in the PE process using engineered RT alternatives to the M‐MLV RT, especially since the wild‐type CaMV RT showed comparable PE efficiency to the engineered M‐MLV RT (Lin *et al*., 2020).

The configuration of the PE protein might alter its efficiency since the fusion of RT to the C‐terminus of nCas9 (H840A) exhibited higher PE activity in human cells (Anzalone *et al*., 2019). However, the N‐terminal fusion of the RT appeared to be better than the C‐terminal fusion for PE activities in rice, potentially because of the alteration of protein expression, stability, folding, or substrate accessibility (Xu *et al*., 2022). The RT enzyme could also be separated from the nCas9 and recruited to the editing sites by the MCP‐MS2 system (Liu *et al*., 2022) (Figure 2b) to improve PE performance. Moreover, to support the activation of nCas9 (H840) during the interrogation process (Sternberg *et al*., 2014), the SpCas9 scaffold sequence was modified (Chen *et al*., 2013), leading to the enhancement of PE activities (Li *et al*., 2022c; Nelson *et al*., 2022).

### Fixing the *de novo* synthesized strand into the genome

The most crucial step that affects PE efficiency is the DNA repair process, which determines whether the RT‐mediated addition of the desired edit is fixed into the targeted genome or eliminated (Figure 1a). After copying genetic information from the RT template, the PE complex left behind a 3′ flap containing the copied sequence (Anzalone *et al*., 2019). Usually, the 3′ terminal of the flap is homologous to the genomic site, thereby competing with the genomic strand for binding and therefore creating an equilibrium between the *de novo* synthesized 3′ ssDNA flap and a genomic 5′ ssDNA flap (Figure 1a), both of which compete to bind to the same site. The heteroduplex formed by the 3′ flap and the complementary target strand thus contains mismatches (Anzalone *et al*., 2022; Chen *et al*., 2021; Ferreira da Silva *et al*., 2022). Nevertheless, the short 5′ flap may be more preferentially excised out by flap endonuclease 1 (FEN1) as it resembles the 5′ ssDNA flaps formed on the Okazaki fragments during lagging strand synthesis of the DNA replication (Ayyagari *et al*., 2003; Sun *et al*., 2023b). The long 5′ flap removal process requires DNA2 nuclease with the assistance of the RPA complex (Bae *et al*., 2001). On the contrary, 3′ flapping enzymes such as TREX1 and TREX2 could interfere with the PE reaction by eliminating the 3′ flaps, as shown in the cases of PE‐mediated sequence insertion (Koeppel *et al*., 2023). If the flap removal is selective to the 5′ flap, the 3′ flap carrying the desired DNA edits could be fixed to the genomic site via one round of DNA replication or targeted strand synthesis triggered by a second nick introduced to it in the PE3/PE3b approach (Anzalone *et al*., 2019; Figures 1 and 2). There would be many other hurdles to the installation of the desired edits to the genome as the MMR machinery is also active, which may recognize the strand containing the edits as erroneous and excise it out (Chen *et al*., 2021; Ferreira da Silva *et al*., 2022; Koeppel *et al*., 2023).

Enhancing the installation of the desired edit‐carrying 3′ flap to the genome was achieved by using the second nick introduced to the nonedit strand to confuse the cellular repair machinery (Anzalone *et al*., 2019). However, the same approach has not seen desired effects in plants, probably due to the variations in RT reactions and subsequent repair mechanisms (Lin *et al*., 2020; Tang *et al*., 2020; Vu *et al*., 2022b; Xu *et al*., 2020a). Another strategy for enhancing desired edit fixing was introducing additional but synonymous mutations to the same‐sense 3′ flap in a PE3 strategy for improving MMR‐mediated removal of the nonedit strand (Li *et al*., 2022d) (Figures 1b and 2c). However, the actual roles of the introduced silent mutations might be evading the MMR as additional mutations altered the mismatch configurations, thereby weakening the mismatch recognition by the Mutator S (*MutS*) proteins (Chen *et al*., 2021; Gupta *et al*., 2012).

Due to the critical role of the MMR in resolving the PE‐introduced sequence that usually leads to the restoration of the original strand, it is likely to enhance PE efficiency by directly suppressing the MMR machinery (Chen *et al*., 2021; Ferreira da Silva *et al*., 2022; Figures 1 and 2b). Knocking out/down the genes encoding for the MMR's components, such as MutS homologue 2 (*MSH2*), *MSH6*, MutL homologue 1 (*MLH1*) and *POSTMEIOTIC SEGREGATION INCREASED 2 (PMS2)*, significantly enhances PE efficiency in human cells (Chen *et al*., 2021; Ferreira da Silva *et al*., 2022). Further, Chen and coworkers developed a new PE version combining PE2/3 with a dominant‐negative *MLH1* (*MLH1dn*) to create PE4/5 versions that showed significant enhancement of PE in human cells (Chen *et al*., 2021; Figures 1 and 2b). However, it might not be the case that *MLH1dn* could work similarly in plants since PE4max did not significantly increase PE efficiency compared with PE2 in rice (Li *et al*., 2022b). Likewise, a tomato dominant‐negative *MSH2* (*MSH2dn*) could not help to enhance PE performance in tomatoes (Vu *et al*., 2022b). These data suggest that either the MMR worked differently with PE in plants or the plant's MMR machinery has evolutionarily been diverged so that the human's *MLH1dn* could not work as efficiently in plants as shown in human cells (Anzalone *et al*., 2022; Li *et al*., 2022b).

### Using paired PEs for escaping from the MMR involvement during desired edit fixation

**Figure 3:**
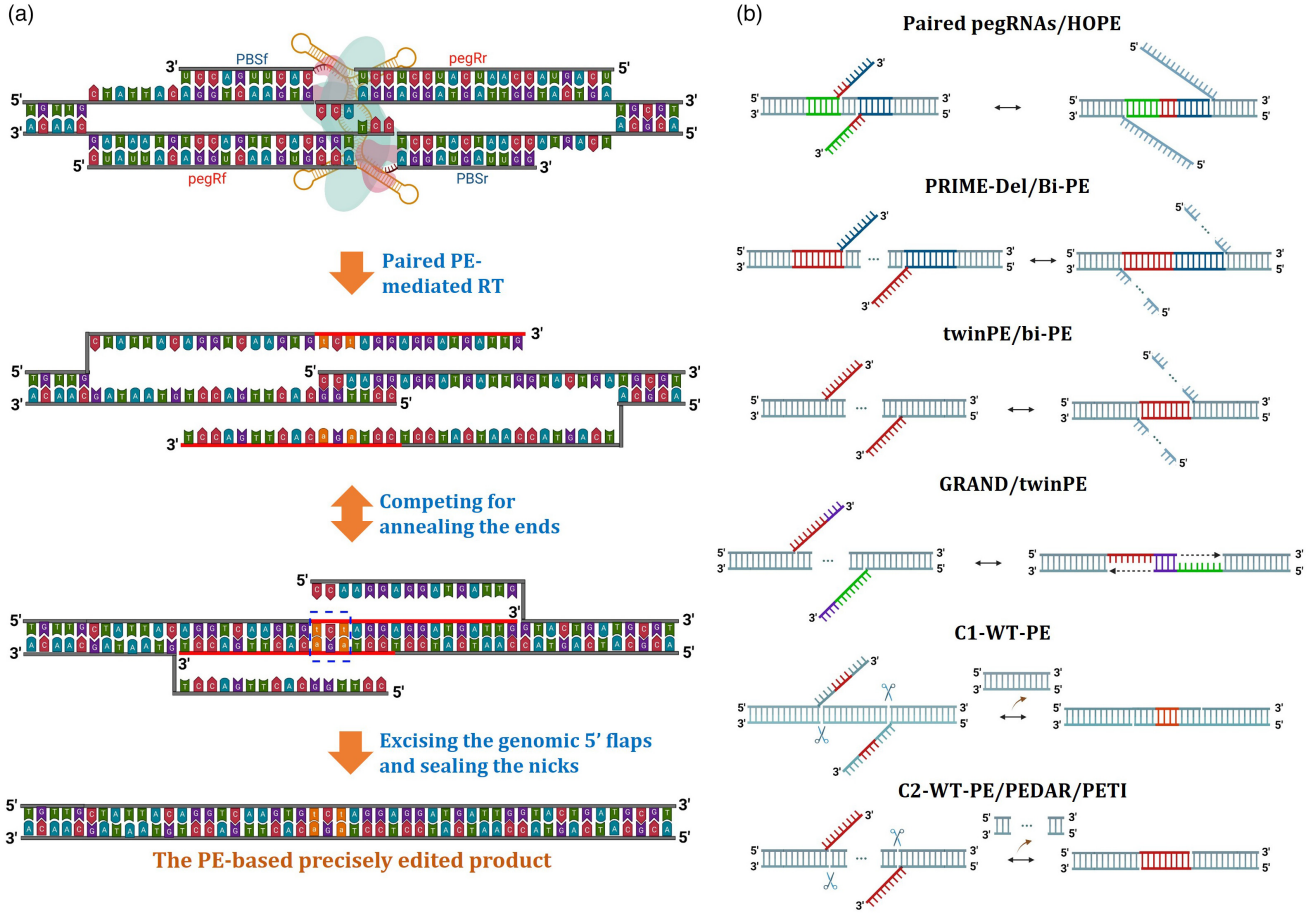
PE approach using paired pegRNAs and major variants. (a) The PE approach utilizes two pegRNAs for targeted editing. Here, we illustrate the target sequence and pegRNAs designed to introduce an herbicide‐resistant allele (P186S: CCA to tCt) into the tomato ALS1 locus. The paired pegRNAs consist of a forward pegRNA (pegRf) and a reverse pegRNA (pegRr), enabling PE on both strands in close proximity. The RT templates are designed to fully complement each other, facilitating annealing and accurate incorporation of the desired base changes (represented by the discontinuous blue box) into the genomic site. (b) Variants of paired pegRNAs. The paired PE variants differ in selecting complementary 3′ extensions, which can be entirely homologous (indicated with the same colour) to the genomic sequence except for the desired base changes, allowing for precise editing in paired PE. Other variants may involve the omission of genomic fragments (PRIME‐Del/bi‐PE) or the omission of genomic fragments while introducing new sequences in the edits (twinPE/PRIME‐Del/bi‐PE/GRAND). In specific cases requiring long‐range DNA fragment deletion, fully functional SpCas9 is utilized for more efficient PE (C1‐WT‐PE and C2‐WT‐PE).

The genius idea was to introduce the pegRNAs in pairs for directly generating 3′ flaps that are entirely complementary with each other using nCas9 (H840A) (Choi *et al*., 2022b; Lin *et al*., 2021; Tao *et al*., 2022b; Wang *et al*., 2022; Zhuang *et al*., 2022) (Figures 2c, 3a,b and Table 2) or fully functional SpCas9 (Jiang *et al*., 2022a; Kweon *et al*., 2023) that potentially omitted the step required the competition of desired edit‐carrying 3′ flap with a 5′ endogenous flap. Using pegRNAs in pairs with the smart arrangement of complementarity between the paired 3′ flaps could significantly enhance overall PE efficiency without elevating by product proportion (Choi *et al*., 2022b; Liang *et al*., 2023; Lin *et al*., 2021; Tao *et al*., 2022b; Wang *et al*., 2022; Zhuang *et al*., 2022) (Figure 3b and Table 2). More importantly, the approach could enable the installation of long insertions and deletions, which are restricted to single pegRNA versions (Chen and Liu, 2023). However, precise insertion or large DNA sequences without altering other bases of targeted sites using paired pegRNAs were still challenging at most tested sites (Lin *et al*., 2021).

**Table 2:** Approaches that used paired pegRNAs for enhancing PE efficiency

No.	Species	Name	Cas protein/RT enzyme	Delivery method	Editing types	Impacts	References
*Human cell lines*							
1	*Homo sapiens*	twinPE	nSpCas9 (H840A)/PE2*	Plasmid and mRNA transfection	Deletion, substitution and insertion of DNA sequences. Chromosomal inversion.	10%–16% insertion efficiency for the 108‐bp fragment with concomitant deletion of 90 bp. 23% for a 46‐bp recoding, 9.4%–27% for a 64‐bp recoding. 28% average efficiency for deleting a 780‐bp sequence containing DMD exon 51. Replaced a 589‐bp sequence with a 38‐bp Bxb1 attB sequence (40% average efficiency). Precise insertion of 5.6‐kb fragment achieved 12%–17% (two steps) and 1.4%–6.8% (one step). Chromosomal inversion efficiency was 7.7%–9.6% and 2.1%–2.6% for sequential and one‐step methods	Anzalone *et al*. (2022)
2	*Homo sapiens*	Bi‐PE	nSpCas9 (H840A)/PE2	Plasmid transfection	Deletions, DNA fragment replacement	Enhanced PE efficiency by up to 16 times and accuracy by 60 times	Tao *et al*. (2022b)
3	*Homo sapiens*	C1‐WT‐PE and C2‐WT‐PE	SpCas9/PE2	Plasmid transfection	Large fragment deletion up to 16.8 megabases (Mb) pairs and inter‐chromosomal translocation	PE efficiency of C1‐WT‐PE (19.1% to 74.0%) outperformed C2‐WT‐PE (8.7% to 61.2%). The chromosomal translocation frequency was ~5% with C1‐WT‐PE and ~4% with C2‐WT‐PE	Tao *et al*. (2022a)
4	*Homo sapiens*	PRIME‐Del	nSpCas9 (H840A)/PE2	Plasmid transfection	Deletions up to 10 kb	1%–30% editing efficiency	Choi *et al*. (2022b)
5	*Homo sapiens*	GRAND	nSpCas9 (H840A)/PE2	Plasmid transfection	Insertion up to 1 kb	63.0% of a 150‐bp insertion with minor by‐products and 28.4% of a 250‐bp insertion	Wang *et al*. (2022)
6	*Homo sapiens*	PEDAR	SpCas9/PE2	RNP transfection and plasmid injection	Simultaneous deletion (up to 10 kb) and insertion (up to 60 bp)	6.97 ± 1.00% (10‐kb deletion and 18‐bp insertion) to 22.6 ± 0.267% (1‐kb deletion and 18‐bp insertion)	Jiang *et al*. (2022a)
7	*Homo sapiens*	PETI	SpCas9/PE2	Plasmid transfection	Chromosomal translocations and inversion	23.9%–43.9% (translocation between HEK3 and HEK4 loci). Chromosomal inversion efficiency was up to 11.6%	Kweon *et al*. (2023)
8	*Homo sapiens*	HOPE	nSpCas9 (H840A)/PE2	Plasmid transfection		Up to 32.6% for insertion, ~35% for deletion	Zhuang *et al*. (2022)
*Plants*							
9	*Oryza sativa* L. ssp. *Japonica*	Dual‐pegRNA	nSpCas9 (H840A) and nSpG‐Cas9 (H840A)/PE2	Plasmid transfection	Point mutations, short DNA deletion and insertion	up to 24.5%	Lin *et al*. (2021)
10	*Oryza sativa* L. ssp*. Japonica*	PrimeRoot	nSpCas9 (H840A), nSpG‐Cas9 (H840A) and nSpRY‐Cas9 (H840A)/PE2	Plasmid transfection	Recombination sequence insertion and long DNA insertion	Recombination sequence (<100 bp) insertion efficiency was up to 50%. Insertion efficiency was 7.9% for a 401‐bp fragment, 2.6% for a 501‐bp fragment and dropped 0.65% for a 720‐bp fragment. The PE‐recombinase‐mediated 5.6‐kb insertion resulted in up to 6.3% efficiency using protoplast transfection and 3.9% efficiency with the *Agrobacterium*‐mediated transformation method	Sun *et al*. (2023a)

* RT from PE2: M‐MLV RT (D200N/L603W/T330P/T306K/W313F).

## PE applications in plants

### Recent progress

#### PE system that utilizes a single pegRNA

Within 1 year of the first PE report (Anzalone *et al*., 2019), the first report showing PE performance in precise editing of all types of single base substitution and short indels at multiple loci in wheat and rice (Lin *et al*., 2020) was also published. Subsequently, many research articles reported PE applications in plants at affordable efficiency (Table 1), albeit they still required improvements. The PE‐mediated edited alleles and phenotypes were stably inherited in offspring of PE‐edited events (Jiang *et al*., 2022b; Qiao *et al*., 2023), even with the complex genomes of hexaploid wheat (Ni *et al*., 2023) indicating the validity and stability of the PE technology and products in plants. Surprisingly, most of the early plant PE works showing efficient PE were conducted in monocots, such as rice, wheat and maize (Butt *et al*., 2020; Hua *et al*., 2020; Jiang *et al*., 2020; Li *et al*., 2020a; Lin *et al*., 2020; Qiao *et al*., 2023; Tang *et al*., 2020; Xu *et al*., 2020a, 2020b). Interestingly, the PE reaction and its consequences may be different from those reported in mammalian cells since introducing second nicks to PE2 did not improve the overall PE efficiency (Hua *et al*., 2020; Jiang *et al*., 2022b; Lin *et al*., 2020; Tang *et al*., 2020; Xu *et al*., 2020a). The PE application progress in dicot plants was slow, possibly due to unexpectedly lower efficiency (Biswas *et al*., 2022; Jiang *et al*., 2022c; Lu *et al*., 2021; Perroud *et al*., 2022; Vu *et al*., 2022b; Wang *et al*., 2021a). The exact reasons have not been revealed besides the possibility of PBS‐spacer autoinhibition and the reduction of Cas9 activity when fused with RT or activated by pegRNAs (Vu *et al*., 2022b).

Although PE2 and PE3/3b resulted in similar PE efficiency in plants, the underlined reasons for the difference to that of the mammalian cells have yet to be revealed. To further improve the PE system in plants, Gao and colleagues attempted to apply various strategies such as PPE (Lin *et al*., 2020) and ePPE (Zong *et al*., 2022), testing temperature impacts and evaluating alternative RT enzymes from bacteria and a plant virus (Lin *et al*., 2020) (Table 1). Subsequently, they were the first to introduce paired pegRNAs and developed a pegRNA designer webpage for facilitating paired pegRNAs selection (Lin *et al*., 2021). Though not all the strategies helped to improve PE performance, the pioneering PE works in plants have inspired many plant groups to study and apply PE for precision plant breeding in monocots (Butt *et al*., 2020; Hua *et al*., 2020; Jiang *et al*., 2020, 2022b; Li *et al*., 2020a, 2022a, 2022b, 2022e, 2023b; Liang *et al*., 2023; Lin *et al*., 2020, 2021; Molla *et al*., 2021; Ni *et al*., 2023; Qiao *et al*., 2023; Tang *et al*., 2020; Wang *et al*., 2021a; Xu *et al*., 2020a, 2021b, 2022; Zou *et al*., 2022), dicots (Biswas *et al*., 2022; Jiang *et al*., 2022c; Lu *et al*., 2021; Perroud *et al*., 2022; Vu *et al*., 2022b; Wang *et al*., 2021a) and moss (*Physcomitrella patens)* (Perroud *et al*., 2022, 2023) (Table 1). Recently, using the PE2max and pU6 composite promoter driving epegRNAs expression (Figure 2c), PE efficiency reached up to 40% in rice, 21.5% in maize and 74.5% in wheat, which are sufficiently high for practical applications in plant breeding (Li *et al*., 2022b; Ni *et al*., 2023; Qiao *et al*., 2023). A similar system using PPE and epegRNAs also vastly improved PE in *P. patens*, albeit only a few loci were tested (Perroud *et al*., 2023).

#### Paired PE approaches

##### Paired PE for short DNA editing

Similar to the data shown in mammalian cells (Anzalone *et al*., 2019), PE efficiency in plants was also shown to be varied among endogenous loci and types of edits (Lin *et al*., 2020). Generally, short‐sequence deletion resulted in the highest PE efficiency, followed by base substitutions (Anzalone *et al*., 2019; Lin *et al*., 2020). Sequence insertion usually results in low efficiency and is dramatically reduced when the insertion length increases (Lin *et al*., 2020). Paired PE systems (Figures 2c, 3 and Table 2) could significantly improve the efficacy and length limitations of PE‐based precise sequence insertion (Liang *et al*., 2023; Lin *et al*., 2021). It was also possible for chromosomal translocation or inversion in human cells (Anzalone *et al*., 2022; Kweon *et al*., 2023). When sequence insertion was designed simultaneously with short sequence deletion at the targeted sites, the efficiency could be significantly higher (Choi *et al*., 2022b; Tao *et al*., 2022b).

##### Paired PE for long DNA modifications

**Figure 4:**
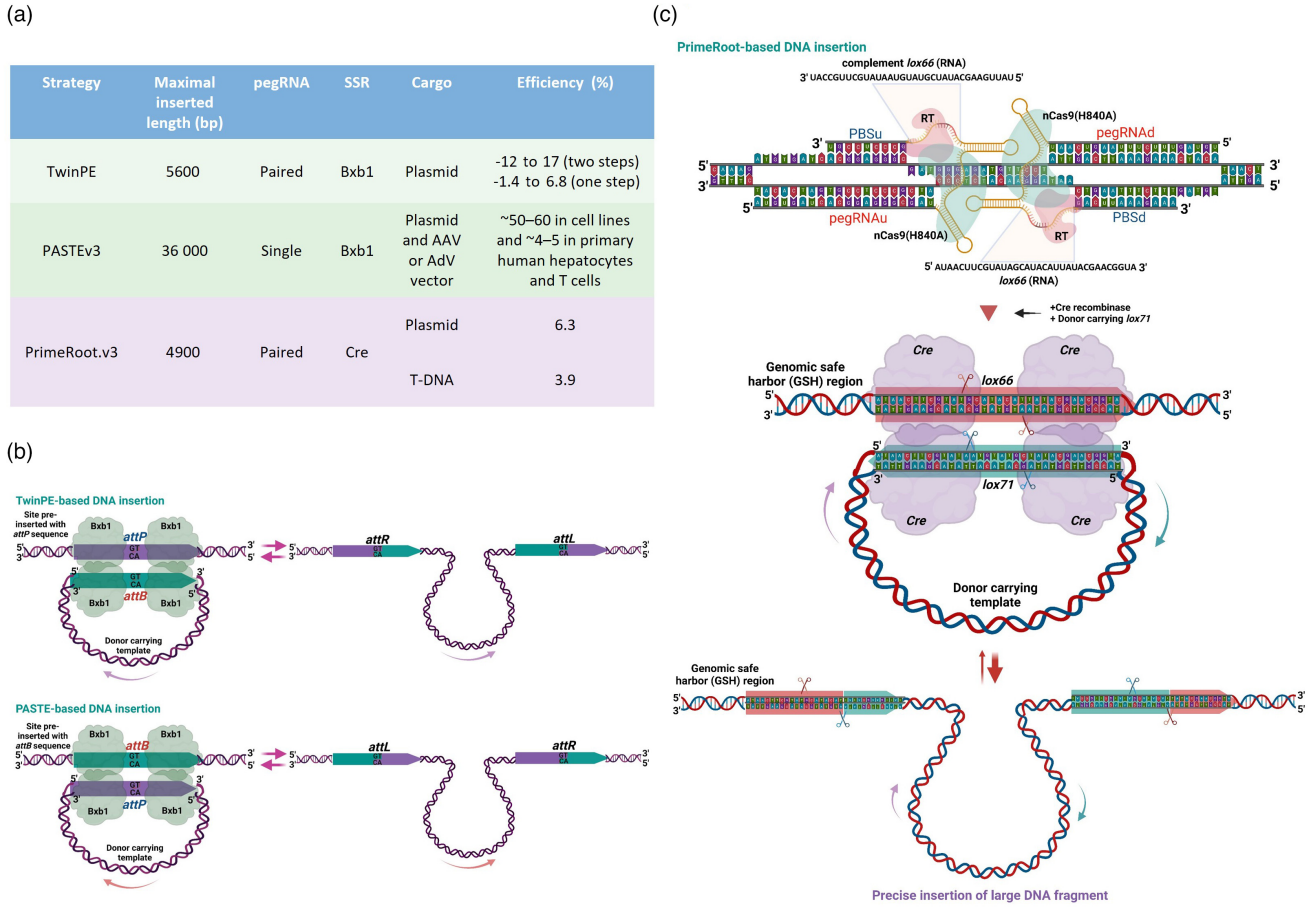
SSR‐mediated precise DNA insertion approaches. (a) Overview of the recently reported SSR‐mediated precise DNA insertion approaches. (b) TwinPE and PASTE‐based DNA insertion using serine SSRs. The TwinPE approach inserted the attP site into the genomic site and placed the attB site within the donor template for recombination. Reversely, the PASTE approach first inserted the attB into the genomic site, and the attP site was used with the donor template for integration. Bxb1 integrases catalyse sequence exchange between the recombination sites (attB with attP or attP with attB), resulting in the insertion of the donor DNA template into the targeted site. (c) Steps involved in PrimeRoot‐mediated DNA insertion using tyrosine SSRs in plants. The PrimeRoot approach utilizes Cre recombinase, a tyrosine recombinase, to mediate DNA insertion at selected safe‐harbour sites within the plant's genomes. Paired pegRNAs are used to instal the *lox66* sequence at the targeted site. A circularized double‐stranded DNA (dsDNA) donor containing the donor DNA template and a *lox71* sequence is introduced. Site‐specific recombination between *lox66* and *lox71* mediated by Cre recombinases, generating nicks (indicated as scissors), results in the precise insertion of the DNA carried by the circularized dsDNA donor.

Gene pyramiding is an important technique to accumulate multiple genes in one plant to improve quality and quantity traits or to simply combine elite traits in one genotype (Servin *et al*., 2004). This approach was beneficial for achieving broad‐spectrum disease resistance in plants (Fuchs, 2017; Fukuoka *et al*., 2015; Pradhan *et al*., 2015; Shehryar *et al*., 2020). However, gene pyramiding via conventional breeding is laborious, time‐consuming and nearly impossible if genes/alleles are linkage‐dragged (Luo *et al*., 2021; Zamir, 2001). Recently, by combining PE and either type of site‐specific recombinases (SSRs) (Merrick *et al*., 2018), kilobase DNA sequences were precisely inserted into predefined genomic sites in mammalian or plant cells (Anzalone *et al*., 2022; Sun *et al*., 2023a; Yarnall *et al*., 2023) (Figure 4a,b). The PE complex was used to insert specific recombination sequences into predefined genomic sites. Subsequently, donor DNAs were precisely inserted into the pre‐installed recombination sites by SSRs (Merrick *et al*., 2018) (Figure 4b) that were simultaneously introduced into the PE‐edited cells (Anzalone *et al*., 2022; Yarnall *et al*., 2023). In plants, a revolutionized paired PE‐based approach called PrimeRoot has enabled the precise insertion of large DNA fragments into a genomic site of choice (Sun *et al*., 2023a). PrimeRoot (Figure 4c) used paired PEs for precisely inserting a recombination sequence such as *lox66* recognized by Cre recombinase (Albert *et al*., 1995; Zhang and Lutz, 2002) (Figure 4c) to theoretically any genomic site of choice, and subsequently, large DNA sequences could be inserted from a donor template carrying a recombination site (*lox71*, Figure 4c) into the site via the activity of a recombinase Cre (Anzalone *et al*., 2022; Sun *et al*., 2023a). Though the technique still requires improvement, PrimeRoot.v3 was successfully employed to generate blast‐resistant rice by precise insertion of a 4.9‐kb expression cassette of a blast‐resistant gene into a safe harbour within the rice's genome (Sun *et al*., 2023a) (Figure 4a). The incredible technology paves a new way to precisely insert genes/alleles of interest into safe harbour sites of plant genomes, which helps to dramatically accelerate precise gene/allele pyramiding and *de novo* domestication in the new plant breeding era.

### The major technical aspects of PE applications in plants

#### Delivery of the PE complex into plant cells

Introducing the PE tool into plant cells is the first step in determining the overall PE efficacy. Plant cells have rigid cell walls, which can impede the delivery of large molecular complexes like the PE system. Therefore, protoplasts would be the first choice to deliver plasmid‐ or RNP‐based PE tools. The first PE report in plants used protoplasts to transfect plasmids encoding PE proteins and pegRNAs (Lin *et al*., 2020). The advantage of this method is the massive load of plasmids or PE RNPs into targeted cells that helps achieve higher PE activity compared to other methods, such as *Agrobacterium*‐mediated transformation. Nevertheless, the major challenge with the protoplast approach is that not all plants have an applicable protocol for efficient and high‐yield protoplast preparation. Moreover, even if the protoplasts were transfected and edited well, not all protoplasts can be regenerated into intact plants (Reyna‐Llorens *et al*., 2023). Though protoplast regeneration could be improved by using various approaches, such as the addition of phytohormones or ectopic expression of regeneration‐related transcription factors, it still requires optimization of the factors in each plant species (Debernardi *et al*., 2020; Reyna‐Llorens *et al*., 2023; Xu *et al*., 2021a). It is also worth noting that there is a limit on the amount and size of plasmid DNA for protoplast delivery (Bart *et al*., 2006; Zhang *et al*., 2011). Particle bombardment appeared suitable for maize transformation but has several issues regarding the insertion of multiple copies of foreign DNAs (Kohli *et al*., 2003) and plant regeneration from largely damaged cells/tissues. The most widely used and cost‐effective method for plant transformation is *Agrobacterium*‐mediated transformation (Gelvin, 2003; Tzfira and Citovsky, 2006). However, agrobacteria could transform well in a range of host plants, but not all. Moreover, this method also requires an efficient plant regeneration protocol to be successfully applied (Altpeter *et al*., 2016). Nevertheless, the *Agrobacterium*‐mediated transformation approach could help to obtain transgene‐free edited lines, thanks to its usually low copy number of transfer DNA (T‐DNA) introduced into targeted cells and thus could be segregated out in the offspring (Gao, 2021), and that could be further improved by inserting T‐DNA into the *Agrobacterium* chromosome (Oltmanns *et al*., 2010). The delivery methods may also reflect the variation in performance of PE in monocots and dicots and, therefore, require careful selection and optimization for each plant species. Finding effective methods to deliver the necessary components into plant cells, particularly in a tissue‐specific or whole‐plant manner, is crucial for successfully applying PE in plants.

#### Editing efficiency and target accessibility

The efficiency of PE can be inconsistent depending on the targeted sites and the specific editing types performed (Li *et al*., 2022b; Lin *et al*., 2020, 2021). One of the possible reasons is the larger size of the PE protein and pegRNA compared to the SpCas9 protein and sgRNA, respectively. Higher PE protein–pegRNA complex sizes possibly pose challenges to target accessibility, particularly the sites occupied by nucleosomes (Horlbeck *et al*., 2016; Yarrington *et al*., 2018). In this case, using ePPE, a plant PPE with a truncated RT enzyme (Jin *et al*., 2023; Zong *et al*., 2022) (Figure 2b) or a split prime editor system (Grünewald *et al*., 2023; Liu *et al*., 2022) for plant PE applications should be considered for high PE performance (Li *et al*., 2022b). Another approach for improving target accessibility is to use a proximal targeting strategy, as shown in rice (Liu *et al*., 2019). A truncated gRNA (14‐15‐nt) can be introduced to the proximal site of a PE‐edited sequence to facilitate R‐loop formation. Moreover, a more robust and optimized configuration of PE proteins such as PEmax (Chen *et al*., 2021) and ePPE (Jin *et al*., 2023; Zong *et al*., 2022) or the most recently updated PE6b, PE6c and PE6d proteins from Liu's group (Doman *et al*., 2023) may be the key to overcoming the PE efficiency barriers in dicot plants since the activity of nCas9‐RT fusion (PE2) was shown to be reduced in tomato (Vu *et al*., 2022b). It is noteworthy that various RT enzymes exhibit distinct behaviours when encountering different types of edits (Doman *et al*., 2023). This observation offers valuable insights into the development of a highly adaptable PE system, enabling the selection of a specialized PE6 variant tailored to specific DNA sequence modifications. In some cases, achieving high editing efficiency can be challenging (Lu *et al*., 2021; Perroud *et al*., 2022; Vu *et al*., 2022b), particularly when attempting to introduce specific edits at genomic loci that are less amenable to modification, such as heterochromatin zones or hypermethylated sites or nucleosome‐occupied sequences (Chen *et al*., 2022; Kim *et al*., 2023). In this case, modifying the genomic contexts might help PE to access and instal desired edits better (Park *et al*., 2021). Further, when oligo T (3–4) is unavoidable within the sequence of the spacer, PBS, or RT template, the RNA polymerase II (RNA‐Pol‐II) promoter would be much better for driving the pegRNA transcription than the RNA‐Pol‐III promoter (Huang *et al*., 2022; Jiang *et al*., 2020; Li *et al*., 2022b). The PBS‐spacer has a self‐complementarity context that significantly reduced Cas9's activities (Ponnienselvan *et al*., 2023; Vu *et al*., 2022b), and thus lowering pegRNAs that could be accessed by the nCas9. In this case, truncating PBS (Ponnienselvan *et al*., 2023) or largely enhancing pegRNA expression by RNA‐Pol‐II promoters such as the pU6 composite (Figure 2c) may help to mitigate the inhibitory effects in dicots (Vu *et al*., 2022b), as recently shown in monocot plants (Li *et al*., 2022b) and moss (Perroud *et al*., 2023). In addition, protecting the 3′ terminal of the pegRNA by secondary structures and altering the pegRNA scaffold (apegRNA) may also add more available pegRNAs for activating PE reactions (Li *et al*., 2022b, 2022c, 2022d; Nelson *et al*., 2022; Perroud *et al*., 2023) (Figure 2c). Moreover, plant PE efficiency could be efficiently improved by using paired PE approaches (Lin *et al*., 2021) (Figure 3). Overall, enhancing the editing efficiency across different species and target sites is an important subject of ongoing PE research in plants.

#### Long DNA sequence modifications

PE allows for various forms of DNA sequence modifications beyond single‐nucleotide changes, such as insertions, deletions and larger scale alterations (Anzalone *et al*., 2019; Chen and Liu, 2023; Lin *et al*., 2020). However, introducing long DNA sequence modifications in plant genomes can be technically demanding (Chen and Liu, 2023; Lin *et al*., 2020; Wang *et al*., 2021a). For sequences less than a kilobase, precise sequence insertions were feasible with paired pegRNAs, albeit at low efficiencies (Lin *et al*., 2021; Zhuang *et al*., 2022) or a template‐jumping approach (Zheng *et al*., 2023). However, efficiently manipulating larger DNA fragments, such as gene insertions or replacements or protein tagging, and ensuring their proper integration into the plant genome pose additional challenges for PE in plants (Lin *et al*., 2020; Wang *et al*., 2021a). Recently, the PrimeRoot strategy (Figure 4c) reported by Gao's group showed the insertion of large DNA sequences at GSH sites at affordable efficiencies (Sun *et al*., 2023a). This technology should also be validated in other plants and may require more optimization for practical applications. Nevertheless, in‐frame kilobase‐scale sequence tagging and gene/allele replacement in plants have not been feasible with PE technology. Recent data has proposed a potential solution for this challenge, known as template‐jumping PE (TJ‐PE). In TJ‐PE, a single TJ‐pegRNA is employed, which carries the in‐frame insertion sequence along with two PBSs. Following the replication of the RT template guided by the TJ‐pegRNA at the 3′ nicked terminus, the newly synthesized sequence, featuring the second PBS at its end, initiates a second RT reaction from the nicked end of the second gRNA (Zheng *et al*., 2023). This approach has been successfully used to insert sequences of up to 800 base pairs in human cell lines (Zheng *et al*., 2023). Incorporating the TJ‐PE approach with the latest and most efficient PE protein architectures, such as PE6 variants (Doman *et al*., 2023), along with utilizing epegRNAs (Li *et al*., 2022b; Nelson *et al*., 2022), could enhance the feasibility of applying this technique in plants (Li *et al*., 2022b; Nelson *et al*., 2022).

#### Off‐target effects

Like other gene editing technologies, PE can induce off‐target mutations, where unintended changes occur in the genome. Fortunately, the PE system uses nickases, single or paired, that usually cause lower levels of off‐targeting (Cho *et al*., 2014; Jin *et al*., 2021; Mikami *et al*., 2016; Zhang *et al*., 2015). Nevertheless, although PE is designed to be highly precise, the occurrence of off‐target effects cannot be completely ruled out (Anzalone *et al*., 2019; Lin *et al*., 2020), especially when paired pegRNAs are used that might mimic DSBs and thus be subjected to more error‐prone repairs. Using additional pegRNAs for individual sites or in pairs also contains more potential off‐target sites or undesired products (Li *et al*., 2023b). Therefore, identifying and minimizing off‐target effects in plant genomes is essential to ensuring the accuracy and safety of PE products (Gao, 2021; Jin *et al*., 2021).

### A recommended experimental design for applying PE techniques in plants

Developing a universally applicable protocol for PE applications across diverse plant species presents a formidable challenge. Nevertheless, in this effort, we aim to provide guidance on essential aspects of a PE experiment that should be considered, irrespective of the specific plant species under investigation. In the context of PE protein selection, the PE2max variant has shown remarkable efficacy in monocots and mosses (Li *et al*., 2022b; Perroud *et al*., 2023). Additionally, the adoption of PE variants, such as ePPE, which reduce the RT enzyme's length by eliminating the RNase H domain, emerges as a promising choice (Zong *et al*., 2022), albeit the overall PE efficiency may be reduced and preferred for pegRNAs with unstructured RT templates (Doman *et al*., 2023; Telesnitsky and Goff, 1993).

For experiments involving plasmid cargoes, it is advantageous to employ a robust promoter–terminator system to govern PE protein expression within the target plant species (de Felippes and Waterhouse, 2023; Diamos and Mason, 2018; Tian *et al*., 2022). When contemplating the use of pegRNA, an engineered variant featuring 3′ terminal protection by tevopreQ1 stands out as the most suitable option (Li *et al*., 2022b; Nelson *et al*., 2022). In the context of enhancing pegRNA transcription, the choice of a U6 composite promoter or a similarly potent promoter is pivotal (Jiang *et al*., 2020; Li *et al*., 2022b).

Furthermore, optimizing the lengths of two pivotal sequences within pegRNA, PBS and RT templates is paramount for achieving peak PE efficiency (Kim *et al*., 2021). In the case of PBS, studies have demonstrated optimal performance at a sequence annealing temperature of approximately 30 °C. Thus, the PBS length may vary between 7 (comprising all C/G) and 15 (comprising all A/U), although recent data suggests that a shorter PBS length may be more effective (Ponnienselvan *et al*., 2023). To commence optimization efforts, a RT template length ranging from 10 to 12 nucleotides provides a suitable starting point (Kim *et al*., 2021). Augmenting PE efficiency could involve the strategic introduction of additional synonymous (silent) mutations at specific sites within the RT template (Li *et al*., 2022d).

For pegRNA designs featuring lengthy PBS and RT template sequences, it is advisable to incorporate RNA chaperones like NC (Zong *et al*., 2022) or consider utilizing the structured RT templates offered by the PE6c and PE6d variants (Doman *et al*., 2023) to ensure proper annealing and enhance RT reaction processivity. When the goal is to insert or replace substantial DNA sequences, the selection of paired PE approaches or the PrimeRoot method represents sound strategies.

## Future perspectives

PE holds tremendous potential for therapeutic applications in human medicine (Anzalone *et al*., 2019, 2020; Chen and Liu, 2023; Doudna, 2020). The precise and versatile nature of PE enables targeted correction of disease‐causing mutations, opening avenues for treating genetic disorders (Anzalone *et al*., 2019, 2020; Chen and Liu, 2023; Doudna, 2020). It offers possibilities for developing personalized medicine approaches and addressing a wide range of genetic diseases that were previously challenging to treat (Chavez *et al*., 2023; Everette *et al*., 2023; Jang *et al*., 2022, 2023; Li *et al*., 2023a).

In plants, PE offers a highly precise and efficient method for introducing specific DNA changes, such as all types of base conversion, DNA sequence insertion or deletion, gene/allele replacement and precise DNA sequence integration in the plant genome (Gao, 2021; Li *et al*., 2023c; Lin *et al*., 2020; Marzec and Hensel, 2020; Sun *et al*., 2023a) (Figures 3 and Tables 1 and 2). It enables the creation of targeted edits without requiring double‐stranded DNA breaks, reducing the potential off‐target effects and undesired mutations (Lin *et al*., 2020). This precision makes PE a valuable tool for precision plant breeding (Li *et al*., 2023c). In addition to introducing nucleotide changes, PE can be harnessed for *in planta* targeted mutagenesis (Xu *et al*., 2021b) and site‐specific gene regulation in plants (Shi *et al*., 2023; Tang and Zhang, 2023). By precisely incorporating designed (known) mutations into non‐coding regulatory regions or promoter elements or upstream ORFs (Liu *et al*., 2021a; Rodríguez‐Leal *et al*., 2017; Shi *et al*., 2023; Tang and Zhang, 2023; Xing *et al*., 2020; Xue *et al*., 2023; Zeng *et al*., 2020; Zhang *et al*., 2018), PE can modulate gene expression levels or alter regulatory sequences as shown with the CRISPR‐Cas9 system. This offers new avenues for fine‐tuning gene expression patterns, enhancing crop productivity and quality, or developing plants with improved stress tolerance (Rodríguez‐Leal *et al*., 2017; Song *et al*., 2022; Wang *et al*., 2021b; Xing *et al*., 2020; Xue *et al*., 2023). Moreover, the PE system can be used to tag endogenous proteins with visible or antigenic markers for *in situ* study of protein localization, functioning and interaction (Choi *et al*., 2022b; Li *et al*., 2023b; Wang *et al*., 2021a; Zong *et al*., 2022), though it requires substantial improvement for routine applications. Further, the PE system and its variants could also be explored for fundamental and applied research in plant synthetic biology, such as those used for recording the transcription history of mammalian cells (Choi *et al*., 2022a), which provides insight into the responses of the plant upon environmental stimulations. Furthermore, PE‐mediated chromosomal translocation/inversion (Anzalone *et al*., 2022; Kweon *et al*., 2023) could be explored for controlling chromosomal recombination and applying it to precision crop breeding, as shown earlier with CRISPR‐Cas9 nucleases (Beying *et al*., 2020; Rönspies *et al*., 2022; Schmidt *et al*., 2020). While the precise implementation of PE‐mediated long DNA modifications in plant breeding is still in its nascent stages (Sun *et al*., 2023a), the continuous development of PE systems, stemming from advancements in both mammalian contexts (Doman *et al*., 2023; Grünewald *et al*., 2023; Nelson *et al*., 2022) and plant research (Jin *et al*., 2023; Sun *et al*., 2023a; Zong *et al*., 2022), remains the driving force behind its improvement. Innovative PE systems, such as the latest PE6 variants (Doman *et al*., 2023), provide a wider array of options for conducting efficient PE experiments in plants. Ultimately, it will help us to design better crops for future agriculture.

By allowing precise modifications in crop genomes, PE can expedite the development of improved crop varieties with enhanced yield, nutritional content, resistance to pests and diseases, herbicide resistance and tolerance to abiotic stresses.

## Concluding remarks

In conclusion, PE is a groundbreaking technique that offers new possibilities for precise genome editing in plants. The PE reaction insight section helps to understand the underlying mechanism of PE, revealing the intricate interplay between the pegRNA, the nCas9/Cas9 variants and the RT enzyme. Understanding the dynamics and requirements of the PE reaction provides valuable insights into optimizing the system for efficient and accurate gene editing in both monocot and dicot plants.

## Funding

This work was supported by the National Research Foundation of Korea (Program 2020M3A9I4038352, 2020R1A6A1A03044344, 2021R1A5A8029490 and 2022R1A2C3010331) and the Program for New Plant Breeding Techniques (NBT, Grant PJ01686702), Rural Development Administration (RDA), Korea.

## Conflict of interest statement

J.Y.K is a founder and CEO of Nulla Bio Inc. The remaining authors declare that the review was written in the absence of any commercial or financial relationships that could be construed as a potential conflict of interest.

## Author contributions

Writing – original draft, T.V.V., N.T.N, J.K. and J.Y.K.; writing – review & editing, T.V.V., J.C.H. and J.Y.K.; funding acquisition, T.V.V., J.C.H. and J.Y.K.; supervision, T.V.V. and J.Y.K. All authors read and approved the manuscript.

## Data Availability

Data sharing is not applicable to this article as no new data were created or analyzed in this study.
